# From Gut to Heart: Havoc in a Young Patient with Typhoid-associated Cardiomyopathy

**DOI:** 10.7759/cureus.5049

**Published:** 2019-07-01

**Authors:** Abdul Majid, Syed Hamza Bin Waqar, Aiman Rehan, Sanjay Kumar

**Affiliations:** 1 Cardiology, Civil Hospital Karachi, Dow University of Health Sciences, Karachi, PAK; 2 Internal Medicine, Civil Hospital Karachi, Dow University of Health Sciences, Karachi, PAK; 3 Internal Medicine, Dow University of Health Sciences, Karachi, PAK

**Keywords:** typhoid, myocarditis, dilated cardiomyopathy, salmonella typhi

## Abstract

Typhoid is an endemic hassle, especially in third-world countries like Pakistan. It is an enteric fever characterized by systemic manifestations that include high temperature and abdominal pain. If not properly treated, at times, it can transgress into complications predominantly involving the gut, where the site of pathology lies. Sometimes, however, it can involve other organ systems and pose diagnostic challenges owing to unfamiliar modes of presentation. Here in, we present a case of a 14-year-old male, previously afflicted and treated for typhoid who presented to the medical consult service with abdominal pain, high-grade fever, and mild chest discomfort. His hemodynamic parameters deteriorated within weeks as he developed pulmonary edema and hypoxemia. He was later diagnosed with echocardiography which earlier on, showed signs of acute myocarditis and eventually dilated cardiomyopathy. The patient was treated with antibiotics coupled with supportive and intensive care which yielded relief in his symptoms. He was later followed up with serial echocardiograms and showed improvement in the cardiac parameters.

## Introduction

Enteric fever, also known as ‘Typhoid,’ is a systemic illness caused by Salmonella enterica serovar typhi, and can be misinterpreted for a number of febrile illnesses. Particularly in developing countries, it is an endemic illness and even the rarest complications can present to clinicians with a variable presentation. Myocarditis and associated cardiomyopathy is one such complication whose prevalence is still not clearly understood [1-2]. Herein, we describe an interesting clinical scenario of a young patient with typhoid who developed cardiac dysfunction secondary to myocarditis and consequent dilated cardiomyopathy.

## Case presentation

A 14-year-old male, previously diagnosed case of typhoid two months back, for which he was admitted in a hospital presented to the medical consult service with ongoing fever for one week and vomiting for three to four days despite the completion of treatment. The fever started one week back and was acute in onset, rising in a stepladder fashion. It was high grade, peaked at 104°F, intermittent in nature, and did not present with any rigors or chills after being administered paracetamol. It was also associated with a constant, generalized and bilateral headache, relieved by pain killers for two to three hours only. The patient also complained of abdominal pain, which he described as diffuse, gradual in onset, aching in character, nonradiating, aggravated by food intake, intermittent and associated with vomiting which occurred just after food intake, three to five times a day. It was around one to two cups per episode, watery in consistency with no particular odor and contained food particles in it. In addition, he had mild chest discomfort which developed after admission and myalgia and fatigue.

On admission, he was fully responsive, alert, and oriented with normal effect. The patient was febrile with a temperature of 104°F, pulse rate of 110 beats per minute (BPM), blood pressure (BP) of 90/60 mmHg, and a respiratory rate of 16 per minute. His abdomen was nondistended, mildly tender with normoactive bowel sounds, and no organomegaly. The neurological exam for bulk, tone, power, and reflexes was insignificant for any finding. Sensations and joint position sense were intact. Cerebellar signs of co-ordination and cranial nerves two to twelve were intact, with no observed nystagmus or visible tremors. The cardiovascular exam showed tachycardia. The cardiac rhythm was normal with no added sounds or murmurs. The pulmonary exam did not yield any abnormal findings, with bilateral audible breath sounds clear to auscultation and no added wheeze. The patient had conjunctival pallor with no lymphadenopathy, edema, jaundice, rashes, or tightening of the skin.

The complete blood count (CBC) showed a hemoglobin (Hb) of 9.8 g/dL and a total leukocyte count (TLC) of 9.7 x 109/L with neutrophils being 82% and lymphocytes being 15%. Platelets were 436 x 109/L. Erythrocyte sedimentation rate was raised up to 84 mm/h (normal: 0-22 mm/h). The coagulation profile was normal as well. The electrolyte panel indicated normal potassium, calcium, and sodium levels. The blood urea nitrogen (BUN) and creatinine were also marginally raised.

The liver function tests (LFT) were within normal limits. Viral serology for hepatitis viruses B and C, dengue virus nonstructural protein 1 (NS1), and dengue IgM were all negative. Coagulation profile was also insignificant. The blood culture showed the growth of Salmonella typhi. The disk diffusion method for susceptibility disclosed the Salmonella typhi to be susceptible to ceftriaxone, cefixime, vancomycin, tazobactam, but resistant to ciprofloxacin, nalidixic acid, amoxicillin, and norfloxacin.

The patient was admitted to the medical inpatient service where he was resuscitated with intravenous fluids. Empirical antibiotic therapy was started with intravenous ceftriaxone. His condition seemed to be improving when on day three, he abruptly developed shortness of breath which worsened gradually. It was associated with orthopnea and paroxysmal nocturnal dyspnea. This raised the suspicion for pulmonary edema and hence, urgent arterial blood gases (ABGs) and chest X-ray (CXR) were ordered.

Arterial blood gases showed a pH of 7.41, partial pressure of oxygen (pO2) of 31.0 mmHg, partial pressure of carbon dioxide (pCO2) of 31.0 mmHg, bicarbonate (HCO3) of 20.7 mmol/L, oxygen saturation (Sat O2) of 49.6%, and base excess (ABE) of -4.0.

Then, the patient was shifted to the intensive care unit (ICU) where he was kept on oxygen support. On examination, he was partially responsive, alert, and oriented with normal effect. The patient was febrile with a temperature of 100°F, pulse rate of 122 BPM, BP of 70/50 mmHg, and respiratory rate of 24/min. His cardiovascular and pulmonary systems were re-examined which showed tachycardia with normal rhythm and a slightly raised jugular venous pressure (JVP). The apex beat was also found to be displaced. On auscultation, there was a third heart sound (S3 gallop rhythm) and bilateral end inspiratory crepitations up to the mid-zone of the posterior chest. During the course of his illness, he also developed bilateral pedal edema.

The CXR showed cardiomegaly with prominent upper lobe vessels and alveolar edema. The costophrenic angles were blunted with right costophrenic angle showing septal lines (Kerley B lines) due to interstitial edema. These findings were highly suggestive of pulmonary edema secondary to heart failure.

Electrocardiography, echocardiography, cardiac enzymes and a Urine Detailed report (Urine D/R) were carried out for further diagnosis.

The cardiac enzymes showed creatine kinase (CK) of 56 IU/L (normal: 20 to 180 IU/L), creatine kinase muscle/brain (CK-MB) of 32 IU/L (normal: 4 to 24 IU/L) and Troponin I of 0.168 of ng/ml (normal: 0 to 0.39 U/L). The Urine detailed report (Urine D/R) showed a marked presence of ketones and proteins.

Electrocardiography (ECG) findings revealed sinus tachycardia with mild QT prolongation and T wave inversions which raised the suspicion for myocarditis.

**Figure 1:**
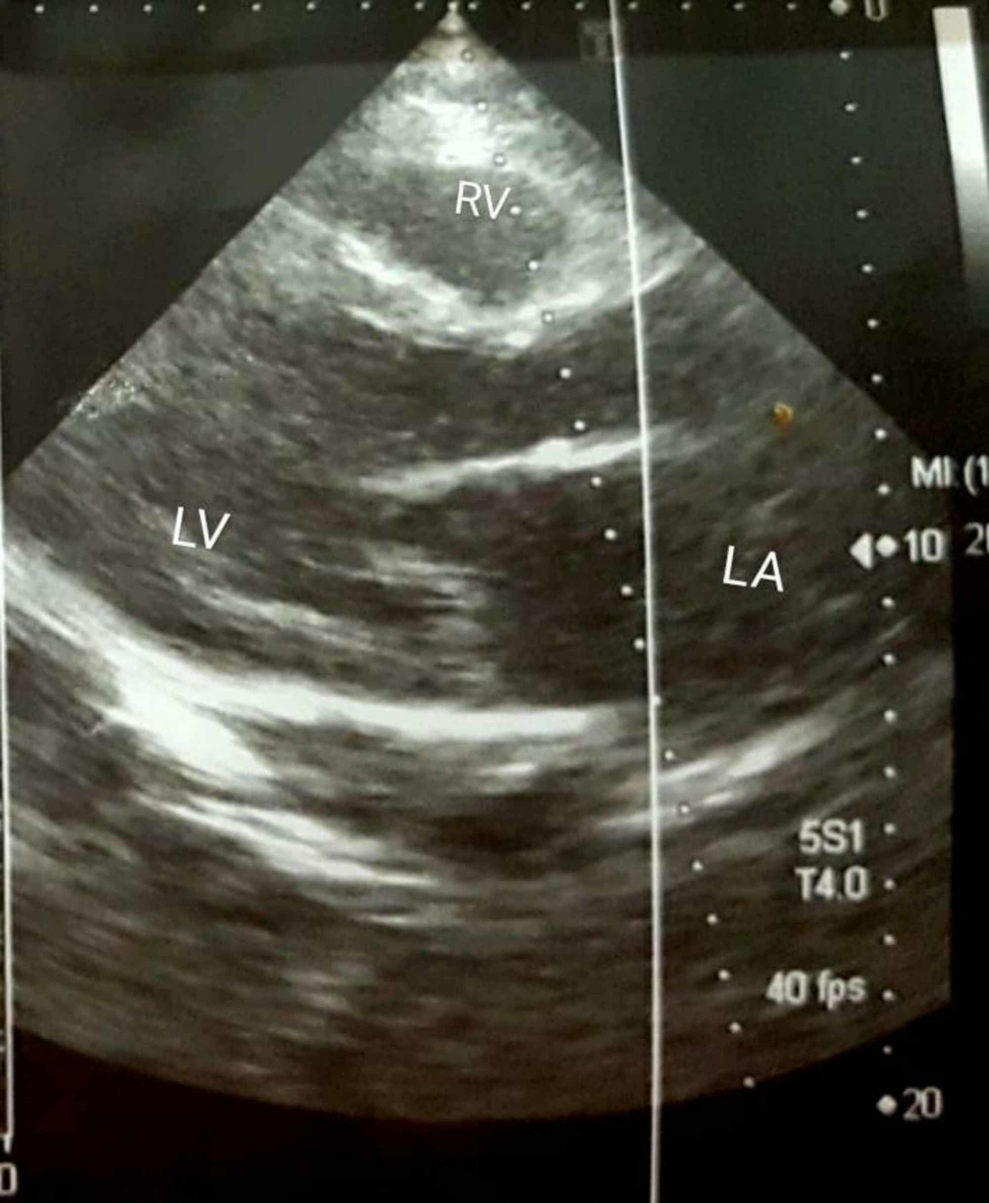
Echocardiogram showing dilatation of LV with wall thinning and mild LA enlargement consistent with dilated cardiomyopathy. LV, left ventricle; LA, Left atrial; RV, right ventricle.

Echocardiography findings revealed an ejection fraction (EF) of 25% with generalized, moderate to severe left ventricular (LV) systolic dysfunction. The left and right ventricles were dilated with right ventricular (RV) dysfunction. ECHO was also significant for mild tricuspid and mitral regurgitation. There was no pericardial effusion or a thrombus in the dilated ventricles. The pulmonary artery systolic pressure was 25 mmHg. These findings were suggestive of dilated cardiomyopathy secondary to myocarditis (Figure 1).

Cardiac magnetic resonance (CMR) imaging and endomyocardial biopsy were not done owing to unavailability of procedures at our set-up and financial constraints.

The patient was then admitted to the critical care unit (CCU) where intravenous furosemide and dexamethasone were given. He was kept on oral enalapril, metoprolol succinate, and spironolactone for five weeks to counter the neurohormonal arcade of myocarditis. Empirical antibiotics were continued with intravenous ceftriaxone for 10 days.

The patient was then discharged after being admitted for three weeks in the CCU and was followed up in medical consults service twice weekly, then once monthly for six months with echocardiography imaging. After six months, his EF improved from 25% to 45% and the patient made a full recovery with no limitations in his daily life activities.

## Discussion

Typhoid is an enteric fever mostly prevalent in third-world countries, contributing to the burden on health care. It is caused by an obligate enteric gram-negative bacillus, Salmonella typhi which has a predilection for the human gut and takes about 14-21 days for incubation inside the gut. Given its fecal-oral route of transmission, it mostly affects the areas with poor hygiene and contributes to an estimated 12 million illnesses and 128,000 deaths on a yearly basis [1]. However, it is striking to know that with the advent of antibiotics, typhoid mortality has dropped down to only 1% from 20% in the pre-antibiotic era. Typhoid is a systemic illness and presents with a multitude of variations which can be diagnostically challenging owing to its nature of involving other organ systems. Even though most of the complications occur in the third week in the absence of antibiotic treatment which mostly results in perforation of the ileum; denying treatment can also cause extra-intestinal problems. These include pulmonary, rheumatological, central nervous system, and hepatobiliary problems. Cardiovascular complications occurred in 1%-5% of patients and include a spectrum of myocarditis and endocarditis as the main complications, and pericarditis and arteritis, which occur less often [1-2].

Myocarditis is an unusual association of typhoid and has only a few reported cases, the first one having been reported in 1884. It is an inflammatory disorder affecting the cardiac muscle and may mimic myocardial ischemia or be totally asymptomatic during its course and then abruptly causing nonspecific hemodynamic instability and symptomatology [3]. Because of the nonspecificity of symptoms, it can be easily missed in the absence of high clinical suspicion [4]. It is important to note that myocarditis can be acute or fulminant and the mode of presentation and prognosis may vary depending on the presentation. Acute myocarditis presents as an indistinct illness leading to gradual hemodynamic alterations and can end up as dilatation and impaired contractility of the ventricles, which is often a bad outcome in the long-term prognosis. On the other hand, fulminant myocarditis presents as a distinct clinical entity with rapid and progressive hemodynamic deterioration but shows a substantial improvement in ventricular function on initiating treatment. Echocardiography can be utilized to differentiate between the two conditions. Patients with fulminant myocarditis had near normal left ventricular diastolic dimensions but increased septal thickness at presentation, while those with acute myocarditis had increased diastolic dimensions but normal septal thickness [5].

Owing to the heterogeneity and rarity of the association of myocarditis and typhoid, the prevalence is not known to date. Interestingly, the mortality associated with myocarditis has been very well established. Cardiovascular insufficiency associated with typhoid myocarditis is the most common cause of death in the second week of illness, with septicemia following its trend [6]. It has been postulated that the pathogenesis of Salmonella typhi is dependent on the amount of inoculum size, virulence, immune-host response, history of previous exposure, and local protective factors [7]. Myocardial damage could be secondary to involvement of the endocardium or due to the direct bacterial invasion from bacteremia. Along with this, sepsis-induced myocardial depression and subsequent remodeling may also play an important role. The most common EKG abnormalities are Q-T prolongation, ST-T changes, bundle branch block, first degree A-V block, and arrhythmias [8].

Echocardiography (ECHO) should initially be done which can help in the detection of impaired ventricular function for the successful diagnosis of myocarditis, even in the subclinical presentations. The findings may include LV dilation, changes in LV geometry for instance development of a more spheroid shape, and wall motion abnormalities. The systolic dysfunction is generally global but may be regional or segmental. An abnormal tissue Doppler signal may provide additional evidence of myocarditis, although data on this is limited. Mild mitral regurgitation and tricuspid regurgitation could also be present [9-10]. Edematous and early-inflammatory and pericardial changes can be visualized in addition to quantification of EF in cases of wall dyskinesis by CMR imaging. CMR findings can help confirm the diagnosis of myocarditis, although sensitivity is variable and time-dependent and abnormalities are nonspecific [11]. The Lake-Louise Criteria for CMR is increasingly used to diagnose clinically suspected myocarditis (Table 1).

**Table 1:** Proposed diagnostic cardiac magnetic resonance (CMR) imaging criteria (Lake Louise Consensus Criteria) for myocarditis.

In the setting of clinically suspected myocarditis, cardiac magnetic resonance (CMR) imaging findings are consistent with myocardial inflammation, if at least two of the following criteria are present:
1. Regional or global myocardial signal intensity increase in T2-weighted images.
2. Increased global myocardial early gadolinium enhancement ratio between myocardium and skeletal muscle in gadolinium-enhanced T1-weighted images.
3. There is at least one focal lesion with nonischemic regional distribution in inversion recovery-prepared gadolinium-enhanced T1-weighted images (late gadolinium enhancement).
‘ A CMR imaging study is consistent with myocyte injury and/or scar caused by myocardial inflammation, if - the third criterion is present.’
A repeat CMR imaging study between one and two weeks after the initial study is recommended, if: - none of the criteria are present, but the onset of symptoms has been very recent and there is strong clinical evidence for myocardial inflammation. - one of the criteria is present.
The presence of left ventricular dysfunction or pericardial effusion provides additional, supportive evidence for myocarditis.

It is increasingly being used against the long-standing gold standard ‘endomyocardial biopsy’ for the diagnosis of myocarditis based on the ‘Dallas Criteria.’ The characteristic features of typhoid fever myocarditis are inflammation of the heart intramural vessels, microcirculatory disturbances, edema and lymphocytic and macrophage infiltration of the stroma, sometimes with formation of granulomas, and dystrophic and necrotic changes of cardiomyocytes [12].

The treatment of typhoid myocarditis and associated cardiomyopathy is no different from other variants of myocarditis. Effective treatment of typhoid with supportive management and antibiotics should be initiated; some studies also show beneficial use of dexamethasone [13]. Non-steroidal inflammatory drugs (NSAIDs) should be best avoided to prevent disruption in the healing of the myocardium. The systolic dysfunction should be countered by blocking the neurohormonal arcade leading to heart failure by initiating angiotensin-converting enzyme (ACE) inhibitors, angiotensin receptor blockers (ARB), beta-blockers, and diuretics. Patients should also be followed up in the long-term to document ventricular recovery with noninvasive imaging [14]. 

## Conclusions

Typhoid disease is an enteric infection, endemic in developing nations like Pakistan and hence, rare complications can crop up and cause peculiar presentations that can prove to be a diagnostic challenge for physicians. Myocarditis and cardiomyopathy can also develop in typhoid, even in immunocompetent cases, and cause an increase in mortality if not appropriately diagnosed and treated. Electrocardiography should be made a regular part of the assessment of typhoid and a low index of suspicion should be kept while considering the diagnosis of myocarditis as it can deteriorate quickly. Serial follow-ups should also be done routinely until the ventricular function returns back to normal.
